# Angiotensinogen Gene Silencing Reduces Markers of Lipid Accumulation and Inflammation in Cultured Adipocytes

**DOI:** 10.3389/fendo.2013.00010

**Published:** 2013-03-11

**Authors:** Wenting X. Carroll, Nishan S. Kalupahana, Suzanne L. Booker, Nalin Siriwardhana, Monique LeMieux, Arnold M. Saxton, Naima Moustaid-Moussa

**Affiliations:** ^1^Department of Animal Science, University of TennesseeKnoxville, TN, USA; ^2^Obesity Research Center, University of TennesseeKnoxville, TN, USA; ^3^Department of Physiology, Faculty of Medicine, University of PeradeniyaPeradeniya, Sri Lanka; ^4^Nutritional Sciences Program, College of Human Sciences, Texas Tech UniversityLubbock, TX, USA

**Keywords:** angiotensinogen, gene silencing, inflammation, adipocytes, adipokines, adipogenesis

## Abstract

Inflammatory adipokines secreted from adipose tissue are major contributors to obesity-associated inflammation and other metabolic dysfunctions. We and others have recently documented the contribution of adipose tissue renin-angiotensin system to the pathogenesis of obesity, inflammation, and insulin resistance. We hypothesized that adipocyte-derived angiotensinogen (Agt) plays a critical role in adipogenesis and/or lipogenesis as well as inflammation. This was tested using 3T3-L1 adipocytes, stably transfected with Agt-shRNA or scrambled Sc-shRNA as a control. Transfected preadipocytes were differentiated and used to investigate the role of adipose Agt through microarray and PCR analyses and adipokine profiling. As expected, Agt gene silencing significantly reduced the expression of Agt and its hormone product angiotensin II (Ang II), as well as lipid accumulation in 3T3-L1 adipocytes. Microarray studies identified several genes involved in lipid metabolism and inflammatory pathways which were down-regulated by Agt gene inactivation, such as glycerol-3-phosphate dehydrogenase 1 (Gpd1), serum amyloid A 3 (Saa3), nucleotide-binding oligomerization domain containing 1 (Nod1), and signal transducer and activator of transcription 1 (Stat1). Mouse adipogenesis PCR arrays revealed lower expression levels of adipogenic/lipogenic genes such as peroxisome proliferator activated receptor gamma (PPARγ), sterol regulatory element binding transcription factor 1 (Srebf1), adipogenin (Adig), and fatty acid binding protein 4 (Fabp4). Further, silencing of Agt gene significantly lowered expression of pro-inflammatory adipokines including interleukin-6 (IL-6), tumor necrosis factor-alpha (TNF-α), and monocyte chemotactic protein-1 (MCP-1). In conclusion, this study directly demonstrates critical effects of Agt in adipocyte metabolism and inflammation and further support a potential role for adipose Agt in the pathogenesis of obesity-associated metabolic alterations.

## Introduction

Obesity is a major risk factor for life-threatening diseases in the United States and worldwide. Its comorbidities include hypertension, cardiovascular disease, stroke, type-2 diabetes mellitus (T2DM), and some types of cancer (Ouchi et al., 2011). In obese individuals, imbalanced secretion of pro- vs. anti-inflammatory adipokines from abnormally expanded adipose tissue and a shift in immune cell populations leads to the development of metabolic complications (Ouchi et al., 2011; Kalupahana and Moustaid-Moussa, 2012a). In the past decades, multiple studies (*in vitro*, *in vivo*, and clinical) indicated the critical role of the renin-angiotensin system (RAS) in the pathogenesis of obesity-associated health disorders. RAS is conventionally known as a group of hormones and enzymes (renin and angiotensin-converting enzyme: ACE) which regulate blood pressure and fluid balance. Angiotensinogen (Agt) is the only known precursor from which the hypertensive hormone angiotensin (Ang) II and other bioactive Ang peptides are generated. Ang II mainly functions through two G-protein coupled receptors, type 1 (AT1) and type 2 (AT2) receptors. In lean individuals, the liver is the major site for Agt production but RAS components are also expressed in white and brown adipose tissue, making them relevant to this work (Karlsson et al., 1998; Engeli et al., 2000; Galvez-Prieto et al., 2008). Angiotensinogen expression in general has been shown to be hormonally and nutritionally regulated as well as differentiation-dependent (Jones et al., 1997a; Siriwardhana et al., 2012). Specifically Agt is thought to be a late marker for adipocyte differentiation (Ailhaud et al., 2002), making it relevant to the current study.

Clinical studies showed that RAS blockade using ACE inhibitors or AT1 blockers improved the insulin sensitivity and lowered the incidence of T2DM in patients with hypertension or other cardiovascular diseases (Pollare et al., 1989). RAS blockade also increased the insulin sensitivity and improved adiposity in obese and diabetic rodent models (reviewed in Kalupahana and Moustaid-Moussa, 2012a). The metabolic effects of RAS were further confirmed in genetic animal models with systemic deletion of certain RAS components (Agt, renin, ACE, AT1a, or AT2 receptor) (reviewed in Kalupahana and Moustaid-Moussa, 2012a). *In vitro* studies showed that Ang II stimulated lipogenesis and secretion of pro-inflammatory adipokines in adipocytes (Jones et al., 1997b; Kalupahana et al., 2012).

In obese humans and rodents, adipose tissue is the major site for Agt production, which significantly increases Agt level in circulation (Van Harmelen et al., 2000; Boustany et al., 2004; Engeli et al., 2005). Our lab and others demonstrated that mice with Agt over-expression in adipose tissue developed obesity with adipocyte hypertrophy, concurrent with insulin resistance and increased expression of lipogenic and pro-inflammatory makers (Massiera et al., 2001a; Kalupahana et al., 2012). Most of these effects were rescued by deletion of AT2 receptor (Yvan-Charvet et al., 2009). The genetic mouse model with adipose-specific Agt gene knock-out exhibited lower systolic blood pressure as they age, however no change was observed in body weight or fat mass when fed a low-fat diet (Yiannikouris et al., 2012). Systemic AGT knock-out mouse models have also been generated in which body weight, adiposity, leptin, and insulin levels were significantly lowered on a high-fat diet compared to wild-type mice. These effects were then reversed when AGT was re-expressed in adipose tissue (Massiera et al., 2001b; Kim et al., 2002).

Studies reviewed above link the elevated secretion of Agt from adipose tissue to obesity-associated local and systemic inflammation as well as insulin resistance, and possibly exacerbated adiposity. Therefore, we hypothesized that inactivation of Agt in adipocytes will limit lipid accumulation, and improve the inflammatory profile.

In the present study, we silenced Agt gene in 3T3-L1 adipocytes using shRNA, and demonstrated that lower Agt expression leads to decreased triglyceride accumulation, which is accompanied by improved expression patterns of adipogenic/lipogenic and inflammatory genes and proteins in adipocytes.

## Materials and Methods

### Cell culture, shRNA transfection, and preadipocyte differentiation

Initially, cell lines were generated as described below using two different shRNA sequences and prepared as both isolated or pooled clones of stably transfected cells. They were then compared to cells stably transfected with scrambled sequences. Both shRNA sequences reduced inflammatory markers and led to significant inactivation of AGT (>70%). Due to the similarities between the two sequences, only one was chosen and used for further detailed experiments as discussed below.

3T3-L1 preadipocytes (American Type Culture Collection; ATCC, Manassas, VA, USA) were seeded in two 6-well cell culture plates. Each well had 2 ml Dulbecco’s modified eagle medium (DMEM) supplemented with 10% fetal bovine serum (FBS). Cells were incubated at 37°C in a humidified CO_2_ incubator. The vector-based shRNA targeting Agt gene (Agt-shRNA, GGATCCCGTTTCTACCTTGGATCCTAGATTGATATCCGTCTAAGGATCCAAGGTAGAAATTTTTTCCAAAAGCTT) was ordered from GenScript (Piscataway, NJ, USA). A scrambled sequence (Sc-shRNA, GGATCCCGTCGCTTACCGATTCAGAATGGTTGATATCCGCCATTCTGAATCGGTAAGCGACGAAGCTTAAGTTTAAACCGCTGATCAGCCTCGACTGTGCCTTCTAGT) with no homology to any mouse or rat mRNA sequence in NCBI database was used as experimental control. These vectors carried a neomycin resistance gene.

Cells were stably transfected at ∼50–60% confluence. The transfection was performed using Lipofectamine™ 2000 Transfection Reagent (Life Technologies, Grand Island, NY, USA) method. 3T3-L1 preadipocytes transfected by Agt-shRNA or Sc-shRNA were maintained in regular growth medium (DMEM containing 10% FBS, 1% penicillin/streptomycin) till 90% confluence. To differentiate the preadipocytes to mature adipocytes, cells were maintained in regular growth medium supplemented by isobutylmethylxanthine (0.5 mM), dexamethasone (0.25 μM), and insulin (1 μg/ml) for 3 days, followed by regular growth media for another 3 days. Several cell lines were generated from these stable transfections and referred to as Agt-ShRNA1, 2, etc. Overall, Agt was silenced by more than 70% in these cell lines.

### Agt and Ang II measurement

Total protein was extracted using tissue lysis buffer (Life Technologies, Grand Island, NY, USA) containing protease inhibitors (Roche, USA). The concentration of extracted protein samples was determined by Bradford assay (Thermo Scientific/Pierce, Rockford, IL, USA).

The expression level of Agt protein was measured by western blotting. Each sample of 25 μg total protein from 3T3-L1 adipocytes transfected by Agt-shRNA or Sc-shRNA (3 vs. 3) was first separated by electrophoresis using 12% Mini-PROTEAN TGX Precast Gel (Bio-Rad, Hercules, CA, USA), and then transferred to a PVDF membrane. After overnight blocking (3% bovine serum albumin in tris-buffered-saline supplemented by 0.1% Tween 20), the membrane was incubated with polyclonal antibodies targeting Agt protein (Santa Cruz Biotechnology, Santa Cruz, CA, USA). After a wash (tris-buffered-saline with 0.1% Tween 20), the membrane was incubated with the secondary antibodies containing horseradish peroxidase. The expression of Agt was detected with the ECL Plus substrate (Thermo Scientific/Pierce, Rockford, IL, USA), and quantified using the NIH ImageJ software. The expression level of Agt protein was normalized by the expression of glyceraldehyde 3-phosphate dehydrogenase (GAPDH).

The intracellular level of Ang II was measured by Angiotensin II EIA Kit (Cayman Chemical, Ann Arbor, MI, USA). To perform the assay, 10 μL of each total protein sample from 3T3-L1 adipocytes transfected by Agt-shRNA or Sc-shRNA (*n* = 5 each) was used. The analyte value was normalized by total protein concentration.

### Lipid accumulation assay

3T3-L1 adipocytes transfected by Agt-shRNA or Sc-shRNA (*n* = 5 each) were washed with PBS, and then harvested in PBS supplemented by 10% IGEPAL CA-630 (detergent, Sigma-Aldrich, St. Louis, MO, USA). Cells were then transferred to a 2 ml autoclaved centrifuge tube and homogenized for 15 s. The triglyceride content in cell lysate was measured using Serum Triglyceride Determination Kit (Sigma-Aldrich, St. Louis, MO, USA). The analyte value was normalized by total protein concentration.

### Pro-inflammatory adipokine profiling

The intracellular level of pro-inflammatory adipokines (IL-1, TNF-α, MCP-1, PAI-1, and resistin) was measured by the Mouse Serum Adipokine Milliplex Kit (Millipore, Billerica, MA, USA). To perform the assay, total protein sample from 3T3-L1 adipocytes transfected by Agt-shRNA or Sc-shRNA (*n* = 5 each) was used. The analyte value was normalized by protein concentration.

### Microarray

Total RNA was extracted from 3T3-L1 adipocytes transfected with Agt-shRNA or Sc-shRNA using RNeasy lipid tissue kit (Qiagen, Valencia, CA, USA). Four sets of RNA samples each were used for microarray analyses. The concentration of these RNA samples was measured using NanoDrop 2000 (Thermo Scientific, USA), and the quality was determined by the Experion RNA StdSens Chips (Bio-Rad, Hercules, CA, USA). Samples were sent to the McGill University’s Génome Québec Innovation Center (Montréal, Canada) and Illumina MouseRef-8 V2.0 Expression Beadchips (Illumina, San Diego, CA, USA) were used to measure 25,697 RefSeq transcripts, over 19,100 genes. David Bioinformatics Database 6.7^1^ (NIAID/NIH) was used for the ontology and pathway analysis on genes with differential expression.

### Quantitative real-time PCR

The expression pattern of selected genes with differential expression in the microarray analyses was verified by quantitative Real-Time PCR (qRT-PCR). Total RNA samples extracted from 3T3-L1 adipocytes transfected with Agt-shRNA or Sc-shRNA (*n* = 4 each) were used for cDNA synthesis using the High Capacity cDNA RT kit (Applied Biosystems, Carlsbad, CA, USA). All primers used in this study were purchased from Qiagen (QuantiTect Primer Assay). The real-time PCR was performed on Applied Biosystems 7900HT Fast Real-Time PCR System using the Fast SYBR Master Mix (Applied Biosystems, Carlsbad, CA, USA). The expression level of housekeeping gene β-actin (Actb) was measured as reference to normalize the expression level of genes under investigation.

### PCR array

The expression of genes associated with adipocyte metabolism was profiled by Mouse Adipogenesis RT^2^ Profiler™ PCR Array (SABiosciences, Qiagen, Valencia, CA, USA), which targets 84 core genes in mouse adipogenesis pathway. The array kit also contains five housekeeping genes, which include Actb. Total RNA samples extracted from 3T3-L1 adipocytes transfected with Agt-shRNA or Sc-shRNA (*n* = 4 each) were used for the synthesis of cDNA using RT^2^ First Strand Kit (SABiosciences, Qiagen, Valencia, CA, USA). RT-PCR was performed on the same equipment as described previously using the RT^2^ SYBR Green qPCR Mastermix (SABiosciences, Qiagen, Valencia, CA, USA). David Bioinformatics Database 6.7 (see text footnote 1, NIAID/NIH) was used for the gene ontology analysis.

### Statistical analyses

In this study, microarray data were analyzed by R software (version 2.9.0) using “lumi” package in bioconductor^2^. Significant differential expression in 3T3-L1 adipocytes with Agt gene silencing compared to the adipocytes transfected by Sc-shRNA was defined as an FDR adjusted *P* value < 0.05.

Data from qRT-PCR and PCR array studies were analyzed by the web-based software^3^ (SABiosciences) using 2^−**ΔΔCT**^ method. The expression level for genes of interest was normalized by the expression of Actb housekeeping gene.

All other analyses were performed using SAS software (version 9.2, Cary, NC, USA). Results were reported as means ± SE. One way ANOVA followed by Tukey *post hoc* test was used to compare the group means at *P* < 0.05.

## Results

### Angiotensinogen gene silencing reduced Agt protein expression and intracellular level of Ang II in 3T3-L1 adipocytes

**Figure 1:**
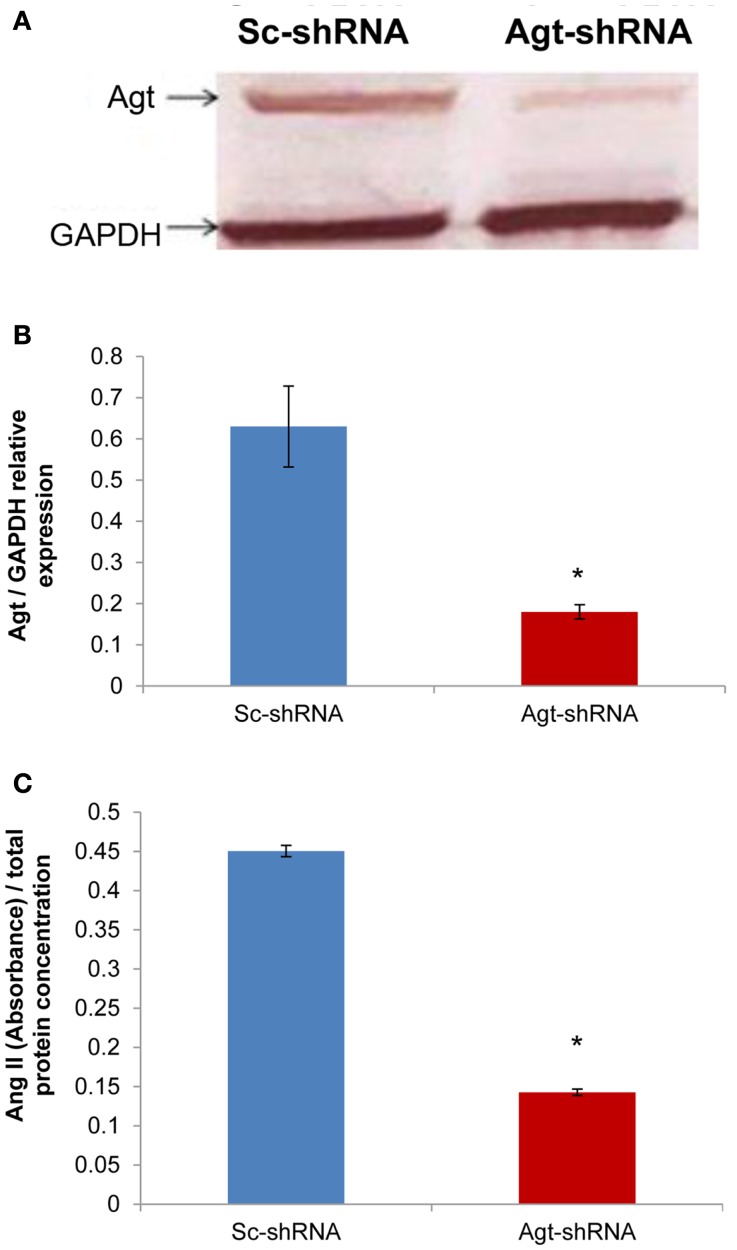
**Agt gene silencing induces lower Agt protein expression and intracellular level of Ang II**. 3T3-L1 preadipocytes were transfected by shRNA targeting Agt gene (Agt-shRNA) or scrambled shRNA (Sc-shRNA), and then differentiated to mature adipocytes. **(A,B)** Agt protein expression, and **(C)** intracellular level of Ang II are shown. Data are presented as mean ± SE [*n* = 3 for each group in **(A)**, *n* = 5 for each group in **(C)**]. The expression of Agt is normalized by the expression of GAPDH. The intracellular level of Ang II is normalized by total protein concentration. *P < 0.005 vs. adipocytes transfected by Sc-shRNA.

Successful transfection of Agt-shRNA into 3T3-L1 preadipocytes was demonstrated by the significantly lower expression of Agt protein in 3T3-L1 adipocytes expressing Agt-shRNA vs. Sc-shRNA (Figures 1A,B; *P* < 0.005). As Agt is the precursor for the hypertensive hormone Ang II, we further measured the intracellular level of Ang II, which was also decreased in 3T3-L1 adipocytes expressing Agt-shRNA vs. Sc-shRNA (Figure 1C; *P* < 0.0001).

### Agt gene silencing decreased lipid accumulation and intracellular level of pro-inflammatory adipokines in 3T3-L1 adipocytes

**Figure 2:**
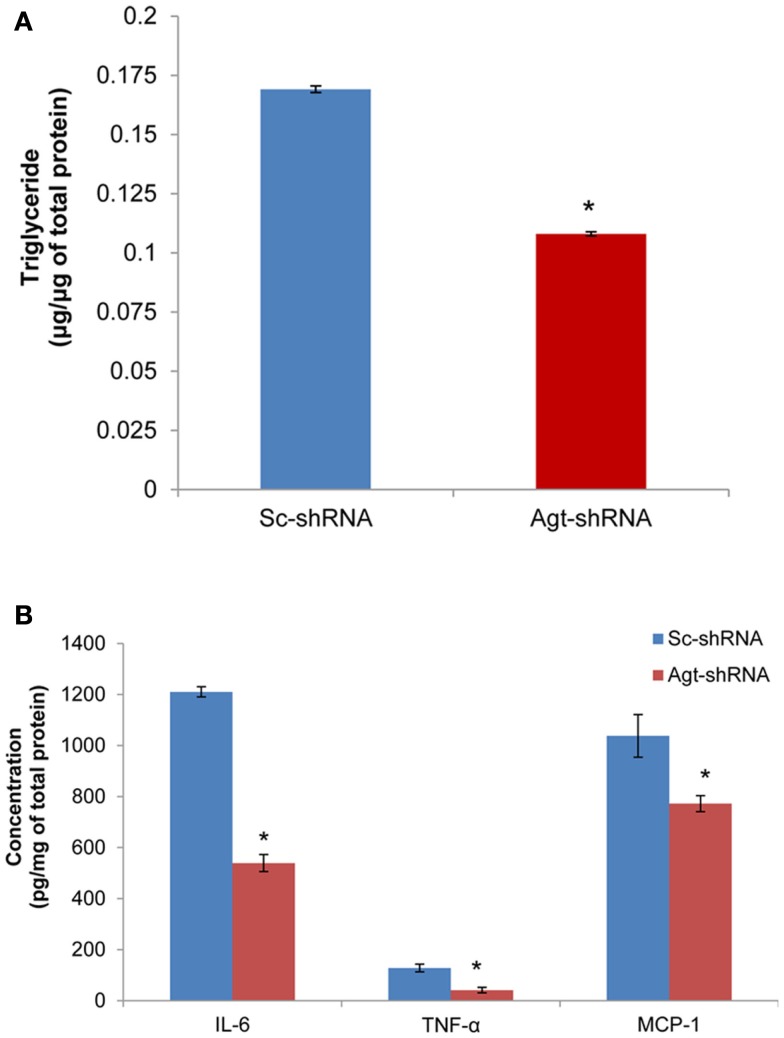
**Agt gene silencing leads to lower triglyceride accumulation and intracellular level of pro-inflammatory adipokines in 3T3-L1 adipocytes**. 3T3-L1 preadipocytes were transfected by shRNA targeting Agt gene (Agt-shRNA) or scrambled shRNA (Sc-shRNA), and then differentiated to mature adipocytes. Triglyceride content **(A)** and adipokine concentration **(B)** is normalized by total protein concentration. Data are presented as mean ± SE (*n* = 5 for each group). *P < 0.05 vs. adipocytes transfected by Sc-shRNA.

Previous studies have reported that Ang II increases the triglyceride content in 3T3-L1 adipocytes (Jones et al., 1997b). Here, we determined the role of RAS in mediating lipogenesis by directly knocking down the expression of Ang II precursor – Agt gene. Agt gene silencing significantly reduced the triglyceride content in 3T3-L1 adipocytes expressing Agt-shRNA vs. Sc-shRNA (Figure 2A; *P* < 0.005). Studies have also shown that Ang II stimulates the secretion of pro-inflammatory adipokines from both human and 3T3-L1 adipocytes. In this study, we measured the intracellular level of major pro-inflammatory adipokines (IL-6, TNF-α, MCP-1, resistin, and PAI-1). The results showed lower IL-6, TNF-α, and MCP-1 protein levels in 3T3-L1 adipocytes transfected with Agt-shRNA vs. Sc-shRNA (Figure 2B). This indicated Agt as an important mediator in adipose inflammation.

### Agt gene silencing induced changes in the expression of genes involved in lipid metabolism and immune response pathways

**Figure 3:**
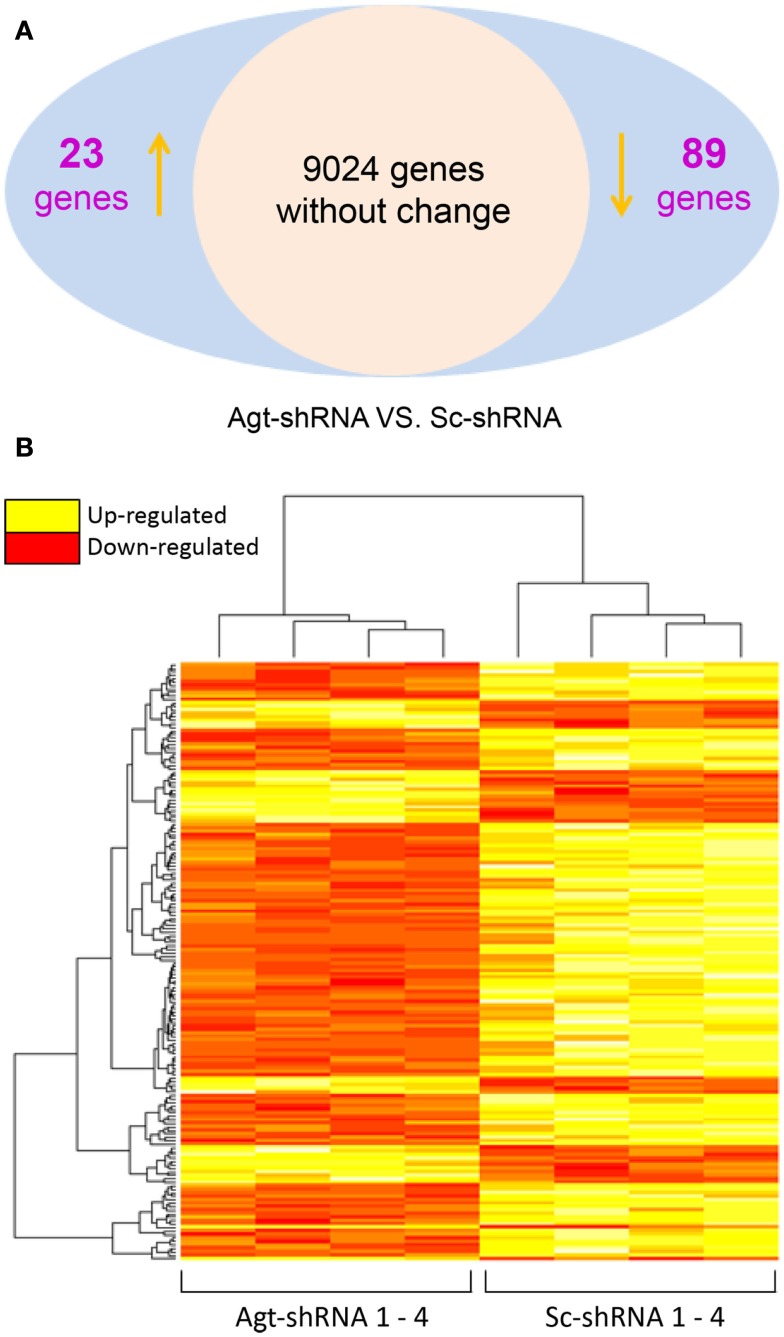
**Agt gene silencing induces changes in global gene expression patterns in 3T3-L1 adipocytes**. 3T3-L1 preadipocytes were transfected by shRNA targeting Agt gene (Agt-shRNA) or scrambled shRNA (Sc-shRNA), and then differentiated into mature adipocytes. Changes in global gene expression patterns were profiled by microarray study. Genes with significant differential expression in 3T3-L1 adipocytes transfected with Agt-shRNA vs. Sc-shRNA were determined by the false discovery rate (FDR) adjusted *P* value < 0.05 **(A)**. Heat map with genes of differential expression is shown in **(B)**. The rows represent the patterns of gene expression, and columns represent samples. Red region corresponds to genes with down-regulated expression, and yellow region corresponds to genes with up-regulated expression, in 3T3-L1 adipocytes transfected with Agt-shRNA vs. Sc-shRNA (*n* = 4 for each cell line).

To dissect the underlying mechanism which mediates the metabolic effects of Agt in adipocytes, a microarray study was performed on 3T3-L1 adipocytes transfected by Agt-shRNA or Sc-shRNA. After filtering out 16,561 transcripts with insignificant expression, we conducted statistical analysis on the remaining 9,136 transcripts. Compared to cells transfected with Sc-shRNA, the expression of 89 genes was significantly down-regulated and the expression of 23 genes was up-regulated in 3T3-L1 adipocytes with Agt gene silencing (Figure 3A). A heat map was plotted by cluster analysis on genes with differential expression in 3T3-L1 adipocytes transfected with Agt-shRNA vs. Sc-shRNA. The expression patterns of these genes were highly consistent within each adipocyte cell line, as shown in Figure 3B.

Gene ontology and pathway analysis showed that the expression of several critical genes involved in lipid metabolism and inflammatory response was down-regulated in 3T3-L1 adipocytes with Agt gene silencing; selected genes are listed in Tables 1 and 2. Other genes with decreased expression in 3T3-L1 adipocytes with Agt gene silencing were genes involved in energy metabolism, immune response, oxidative stress, cell differentiation, and apoptosis. Genes with up-regulated expression in 3T3-L1 adipocytes with Agt gene silencing were ones involved in DNA replication, transcription, translation, repair, chromosomal structure, intracellular protein trafficking, protein phosphorylation, and proteolysis. These genes are listed in Table A1 in Appendix.

**Table 1:** **Lipid metabolic genes differentially expressed in 3T3-L1 adipocytes transfected by Agt-shRNA vs. Sc-shRNA**.

Gene symbol	Gene name	Fold change	*P* value (FDR adjusted)	Function
Gpd1	Glycerol-3-phosphate dehydrogenase 1	0.34	0.003919	A lipogenic marker, catalyzes the reversible conversion of dihydroxyacetone phosphate to sn-glycerol-3-phosphate
Ces3	Carboxylesterase 3	0.24	0.000087	Adipocyte lipase, mediates lipolysis
Retsat	Retinol saturase	0.63	0.025349	Saturates13-14 double bond of all-trans-retinol to produce all-trans-13, 14-dihydroretinol
Acad10	Acyl-Coenzyme A dehydrogenase family, member 10	0.78	0.050300	Catalyzes the initial step of mitochondria fatty acid β-oxidation
Apoc1	Apolipoprotein C-I	0.48	0.001845	Modulates the interaction of APOE with beta-migrating VLDL, inhibits the binding of beta-VLDL to LDL receptor-related protein
Hsd3b7	Hydroxy-delta-5-steroid dehydrogenase, 3 beta-, and steroid delta-isomerase 7	0.80	0.048719	Plays a crucial role in the biosynthesis of hormonal steroids
Suclg1	Succinate-CoA ligase, GDP-forming, alpha subunit, mitochondrial	0.72	0.037140	An enzyme in tricarboxylic acid cycle, catalyzes ATP- or GTP- dependent ligation of succinate and CoA to form succinyl-CoA in mitochondrial matrix

*FDR, false discovery rate*.

**Table 2:** **Inflammatory genes differentially expressed in 3T3-L1 adipocytes transfected by Agt-shRNA vs. Sc-shRNA**.

Gene symbol	Gene name	Fold change	*P* value (FDR adjusted)	Function
Saa3	Serum amyloid A 3	0.57	0.025314	Major acute phase protein, in response to cytokine stimulation
Nod1	Nucleotide-binding oligomerization domain containing 1	0.75	0.029593	Induces NF-κ B activation via RIPK2 and IKK-gamma, enhances caspase-9-mediated apoptosis
Cxcl12	Chemokine (C-X-C motif) ligand 12	0.38	0.046402	Chemoattractant for T-lymphocytes and monocytes
Ptx3	Pentraxin related gene	0.71	0.033282	Activates classical complement pathway, facilitates pathogen recognition by immune cells
Stat1	Signal transducer and activator of transcription 1	0.66	0.029154	A member of signal transducers and activators of transcription (STATs) family, mediates cytokine signaling
Irf9	Interferon regulatory factor 9	0.64	0.029593	Plays a critical role in stimulating IFN-alpha responsive genes

*FDR, false discovery rate*.

**Figure 4:**
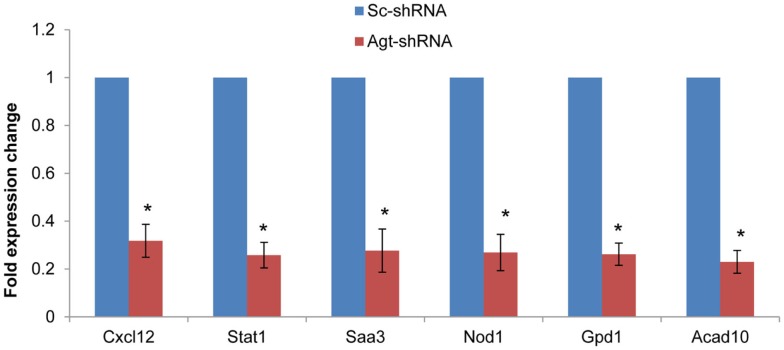
**Agt gene silencing induces down-regulated expression of Cxcl12, Stat1, Saa3, Nod1, Gpd1, and Acad10 genes in 3T3-L1 adipocytes**. 3T3-L1 preadipocytes were transfected by shRNA targeting Agt gene (Agt-shRNA) or scrambled shRNA (Sc-shRNA), and then differentiated to mature adipocytes. Data are presented as mean ± SE, and calculated according to the 2^−**ΔΔCT**^ method (*n* = 4 for each group). *P < 0.0001 vs. adipocytes transfected by Sc-shRNA.

To determine whether the profile of global gene expression from the microarray study were consistent with the relative amounts of mRNA present in parallel samples, qRT-PCR was performed on selected genes (Cxcl12, Stat1, Saa3, Nod1, Gpd1, and Acad10). Consistent with microarray results, the expression level of all these genes were down-regulated in 3T3-L1 adipocytes with Agt gene silencing compared to those transfected with Sc-shRNA (Figure 4).

**Figure 5:**
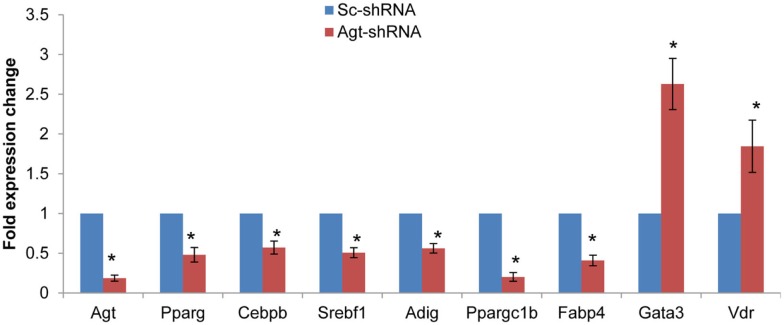
**Agt gene silencing induces changes in the expression of genes associated with adipogenesis pathway in 3T3-L1 adipocytes**. 3T3-L1 preadipocytes were transfected by shRNA targeting Agt gene (Agt-shRNA) or scrambled shRNA (Sc-shRNA), and then differentiated to mature adipocytes. Data are presented as mean ± SE, and calculated according to the 2^−**ΔΔCT**^ method (*n* = 4 for each group). *P < 0.05 vs. adipocytes transfected by Sc-shRNA.

Since knock-down of Agt gene decreased lipid accumulation and the expression of genes associated with lipid metabolism in 3T3-L1 adipocytes, we further profiled the expression pattern of genes in the adipogenesis pathways using a PCR array. The results confirmed the lower expression of Agt gene at transcription level, and showed decreased expression of several adipogenic genes, including PPAR-γ, Cebpb, Srebf1 and Adig, and lipid metabolic markers Ppargc1b and Fabp4 in 3T3-L1 adipocytes with Agt gene silencing. Conversely, the expression of genes with anti-adipogenic effects, Gata3, and Vdr, were up-regulated. The expression pattern of these genes is shown in Figure 5, and the functions of these genes are described in Table 3.

**Table 3:** **Genes with differential expression associated with adipogenesis pathway in 3T3-L1 adipocytes transfected by Agt-shRNA vs. Sc-shRNA**.

Gene symbol	Gene name	Fold change	*P* value	Function
Pparg	Peroxisome proliferator activated receptor gamma	0.4808	0.030656	Key regulator of glucose homeostasis and preadipocyte differentiation
Cebpb	CCAAT/enhancer binding protein (C/EBP), beta	0.5718	0.038756	Plays a catalytic role in preadipocyte differentiation
Srebf1	Sterol regulatory element binding transcription factor 1	0.5070	0.008977	Regulates glucose metabolism, fatty acid, and lipid (mainly cholesterol) synthesis
Adig	Adipogenin	0.5619	0.005956	An adipocyte-specific protein, promotes adipocyte differentiation
Ppargc1b	Peroxisome proliferative activated receptor, gamma, coactivator 1 beta	0.2017	0.045250	Stimulates the activation of transcription factors. Involved in fat oxidation, and non-oxidative glucose metabolism
Fabp4	Fatty acid binding protein 4	0.4094	0.002358	Adipocytes specific lipid transport protein
Gata3	GATA binding protein 3	2.6291	0.003210	Suppresses preadipocytes differentiation through PPAR-γ
Vdr	Vitamin D receptor	1.8460	0.003185	Suppresses preadipocytes differentiation through C/EBP and PPAR-γ

## Discussion

Recent studies indicated the potential role of adipose tissue-derived Agt in the pathogenesis of metabolic disorders. However, the underlying mechanisms mediating these effects are not well clarified. In the present study, we successfully established Agt gene silencing in 3T3-L1 adipocytes using shRNA, which effectively decreased the intracellular level of Agt and Ang II, and enabled us to directly investigate the metabolic effects of adipose Agt. Using this *in vitro* model, we confirmed the direct contribution of adipocyte-derived Agt and Ang II to preadipocyte differentiation, lipogenesis, and adipocyte inflammation, as evidenced by decreased triglyceride accumulation, and pro-inflammatory adipokine production, as well as down-regulated expression of several adipogenic and inflammatory genes.

### RAS and adiposity

The present study confirmed the effects of Agt/Ang II in promoting lipogenesis. Our lab previously reported that Ang II increases lipogenesis in 3T3-L1 and human adipocytes (Jones et al., 1997b). In line with these findings, we demonstrated in this study that inactivation of Agt gene significantly reduced the lipid accumulation during 3T3-L1 preadipocyte differentiation. This is also in agreement with a recent study where shRNA-mediated Agt gene silencing inhibited triglyceride storage during human visceral preadipocyte differentiation (Ye et al., 2010). Indeed, in these studies, the expression of Gpd1 gene which encodes glycerol-3-phosphate dehydrogenase 1 was increased in Ang II treated 3T3-L1 adipocytes (Jones et al., 1997b) but decreased by knockdown of the Agt gene in human preadipocytes. Consistently, we identified here that the expression of Gpd1 gene was decreased in 3T3-L1 adipocytes with Agt gene silencing. In this study, no change was detected in the gene expression of acetyl-coenzyme A carboxylase or fatty acid synthase, the major enzymes mediating *de novo* lipogenesis. Therefore, the lower lipid accumulation in 3T3-L1 adipocytes with Agt gene silencing is potentially due to the decreased synthesis of glycerol by Gpd1 in the process of lipogenesis. It is also possible that Agt gene silencing down-regulates the activity of lipogenic enzymes, and inhibits the *de novo* lipogenesis in 3T3-L1 adipocytes. Ang II was previously shown to inhibit preadipocyte differentiation, which was potentially mediated by the AT1 receptor. Similarly, certain AT1 blockers enhanced preadipocyte differentiation in both 3T3-L1 and human preadipocytes, and mice with systemic knock-out of AT1a receptor showed adipocyte hypotrophy (Janke et al., 2002, 2006; Schupp et al., 2004). However, in this study, we identified that Agt gene silencing inhibited 3T3-L1 preadipocyte differentiation through down-regulating the expression of core adipogenic genes (C/EBP-β and PPAR-γ) and other adipogenic markers (Srebf1 and Adig) which mediate the initiation of adipogenesis. Agt gene silencing also decreased the expression of several lipid metabolic genes (Fabp4 and Ppargc1b). Genes with anti-adipogenic effects (Gata3 and Vdr) were also up-regulated in Agt-shRNA transfected cells. These indicated Agt as a critical factor with stimulatory effects on preadipocyte differentiation. We propose that the effect of Ang II on adipogenesis is receptor specific, and dose dependent. Possibly, low to regular levels of Ang II stimulate adipogenesis through the AT2 receptor, which overcomes the anti-adipogenic effect mediated by the AT1 receptor. However, high levels of Ang II could activate the AT1 receptor and inhibit preadipocyte differentiation.

Our microarray study detected multiple genes associated with adipocyte metabolism which were down-regulated by Agt gene silencing. Retsat (encodes retinol saturase) is induced during 3T3-L1 preadipocyte differentiation and promotes adipogenesis through increased PPAR-γ transcriptional activity (Schupp et al., 2009).

Ang II is an active mediator of lipolysis in a time-dependent manner. Short term treatment of Ang II inhibits lipolysis in human adipose tissue and adipocytes through the AT1 receptor (Boschmann et al., 2001; Goossens et al., 2004, 2007). However, long term infusion of Ang II markedly increased lipolysis in rats (Cabassi et al., 2005). One explanation for this could be the Ces3 gene which encodes for carboxylesterase 3 (triacylglycerol hydrolase, TGH) – an adipocyte lipase – whose expression was decreased in 3T3-L1 adipocytes with Agt gene silencing.

Lastly, our microarray and RT-PCR data indicated that Agt gene widely affects the whole process of lipid metabolism in adipocytes. This was seen in the expression of other genes down-regulated by Agt gene silencing, such as Acad10, Apoc1, Suclg1, and Hsd3b7, which are involved in lipid transport, β oxidation, TCA cycle, and biosynthesis of steroid hormones from lipids.

### RAS and inflammation

While it has been established that obesity leads to chronic low-grade inflammation in adipose tissue (Kalupahana and Moustaid-Moussa, 2012a), the exact trigger for this process is unknown. Adipose tissue-secreted Agt has been shown to induce obesity-associated inflammation, a major factor for the pathogenesis of metabolic disorders, and therefore is a potential target for obesity research as described below (Kalupahana and Moustaid-Moussa, 2012b). In obesity, adipose tissue-derived Agt significantly increases the level of Agt in circulation (Van Harmelen et al., 2000; Boustany et al., 2004; Engeli et al., 2005). Moreover, our lab and collaborators further showed that adipose-specific Agt over-expression induced local and systemic inflammation, as well as insulin resistance in mice (Kalupahana et al., 2012).

Previous studies reported that Ang II stimulated the secretion of several pro-inflammatory adipokines from both 3T3-L1 and human adipocytes (Skurk et al., 2001, 2004). Consistent with those studies, we demonstrated here that Agt gene silencing in 3T3-L1 adipocytes significantly reduced the intracellular level of pro-inflammatory adipokines, including MCP-1, IL-6, and TNFα.

In the current research, the microarray study identified that Agt gene silencing decreased the expression of several genes involved in adipose inflammation including Saa3, Nod1, Stat1, and Cxcl12. Saa3, which encodes for the acute phase protein – serum amyloid A3, was recently identified as a critical pro-inflammatory adipokine involved in obesity-associated metabolic disorders (Yang et al., 2006). A previous study showed that blocking the AT1 receptor suppressed the mRNA expression of Saa3, together with TNF-a, PAI-1, and MCP-1 in mice adipose tissue (Kurata et al., 2006). The present study further confirmed that Saa3 as a critical factor involved in Ang II-induced inflammatory disorders, as silencing of Agt in cultured adipocytes reduced Saa3 expression.

Nod1 gene encodes an intracellular pattern recognition receptor mediating immune response. The activation of this receptor induces peripheral and hepatic insulin resistance, which is prevented when this gene in inactivated (Schertzer et al., 2011). Activation of NOD1 also increases pro-inflammatory adipokine secretion and impairs the insulin-stimulated glucose uptake in 3T3-L1 and primary murine adipocytes (Schertzer et al., 2011; Zhao et al., 2011). Since Agt silencing reduced NOD1, this suggests that Nod1 is a potential mediator for Ang II-induced adipocyte inflammation.

Stat1 is a member in the Stats family – the major component of Jak-Stat pathway which plays a critical role in mediating cytokine signaling. Jak-Stat pathway mediates the action of hormones which impact adipocyte development and functions (Richard and Stephens, 2011). Stats mediate the activation of multiple inflammatory pathways, such as NF-κB and c-Jun (O’Shea et al., 2002). The lowered expression of the Stat1 gene, in part, indicates that Agt gene silencing down-regulates the cytokine signaling in 3T3-L1 adipocytes. Interferon regulatory factors (Irfs), which mainly respond to interferon in the JAK-STAT signaling pathway (O’Shea et al., 2002; Eguchi et al., 2008), could be one avenue of this down-regulation. Previous studies reported that IFN-α/β induced a complex formation of STAT1, STAT2, and IRF9, one of the Irfs which are expressed in adipocytes (Eguchi et al., 2008). Together these indicate that Agt gene silencing induced a consistent change pattern in cytokine signaling pathway (O’Shea et al., 2002; Eguchi et al., 2008).

Several other chemokines were altered by Agt inactivation. These include Cxcl12 and Ptx3. Cxcl12 is mainly expressed in stromal cells, but is also detected in 3T3-L1 adipocytes (Choi et al., 2010). This indicates the potential role of this gene in inducing the immune cells infiltration in adipose tissue. The expression of Ptx3 gene is up-regulated during preadipocyte differentiation, and although almost non-detectable in mature adipocytes, it can be re-activated in 3T3-F442A adipocytes upon TNF-α stimulation (Abderrahim-Ferkoune et al., 2003). The decreased expression of Ptx3 gene in 3T3-L1 adipocytes with Agt gene silencing is potentially mediated by decreased adipogenesis and TNF-α (Abderrahim-Ferkoune et al., 2003).

Elevated level of RAS components was detected in adipose tissue and circulation in obese human subjects and animals (Van Harmelen et al., 2000; Boustany et al., 2004; Engeli et al., 2005). Animal models with systemic deletion of RAS components (Agt, renin, ACE, AT1a, and AT2) were protected from diet-induced obesity and metabolic disorders (Massiera et al., 2001b). Transgenic mice with Agt over-expression in adipose tissue exhibit adiposity, adipocyte hypertrophy, adipose and systemic inflammation, and insulin resistance (Kalupahana et al., 2012). However, mice with Agt gene specifically knock-out from adipose tissue had no change in fat mass or body weight (Yiannikouris et al., 2012). These indicate that elevated secretion of Agt from adipose tissue may in part mediate obesity-associated metabolic disorders.

**Figure 6:**
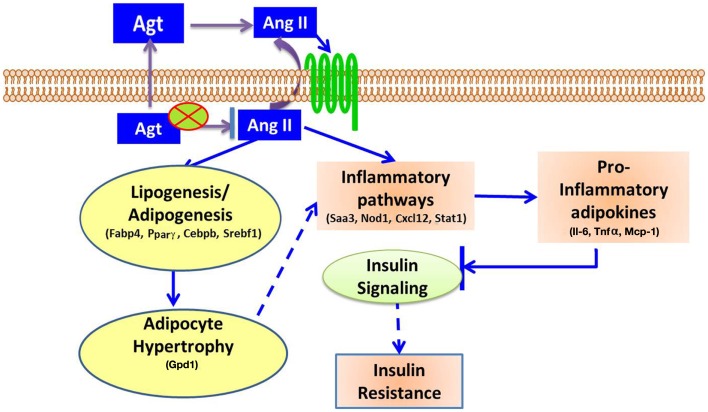
**Proposed mechanisms for Ang II-induced metabolic disorders**. Adipocyte-derived Agt is converted to Ang II which affects adipocytes in autocrine or paracrine manner through angiotensin receptors. Ang II activates inflammatory pathways, and increases the secretion of pro-inflammatory adipokines, which inhibit insulin signaling and induce insulin resistance. Ang II also stimulates lipogenesis in adipocytes, leading to adipocyte hypertrophy which potentially activates inflammatory pathways and exacerbates these inflammatory conditions.

In summary, this study showed that gene silencing induced lower adipocyte-derived-Agt, which led to decreased lipid accumulation and pro-inflammatory adipokines production, as well as down-regulated expression of adipogenic and lipid metabolic markers and genes in inflammatory pathways. The underlying mechanism for the metabolic effect of Ang II is summarized in Figure 6.

## Conflict of Interest Statement

The authors declare that the research was conducted in the absence of any commercial or financial relationships that could be construed as a potential conflict of interest.

## Footnotes

^1^http://david.abcc.ncifcrf.gov/tools.jsp

^2^http://www.bioconductor.org/

^3^http://pcrdataanalysis.sabiosciences.com/pcr/arrayanalysis.php

## Appendix

**Table A1:** **Genes differentially expressed in 3T3-L1 adipocytes transfected with Agt-shRNA vs. Sc-shRNA**.

Gene symbol	Gene name	Fold change	*P* value (FDR adjusted)
**ENERGY METABOLIC PROCESS**			
Por	P450 (cytochrome) oxidoreductase	0.613868	0.004184
Aldh2	Aldehyde dehydrogenase 2, mitochondrial	0.656561	0.022585
Cdo1	Cysteine dioxygenase 1, cytosolic	1.806253	0.041216
Cyb561	Cytochrome b-561	0.661127	0.029593
Idh3g	Isocitrate dehydrogenase 3 (NAD+), gamma	0.65022	0.021874
Hibadh	3-hydroxyisobutyrate dehydrogenase	0.729005	0.033282
Phgdh	Phosphoglycerate dehydrogenase	0.688725	0.035963
gm2a	GM2 ganglioside activator protein	0.511569	0.029154
AU018778	Expressed sequence AU018778 (type-B carboxylesterase/lipase)	0.439825	0.000356
Atp6ap1	ATPase, H+ transporting, lysosomal accessory protein 1	0.666187	0.031342
Acaa1b	Acetyl-Coenzyme A acyltransferase 1B	0.627201	0.031219
Adhfe1	Alcohol dehydrogenase, iron containing, 1	0.669428	0.029465
Gpt	Glutamic pyruvic transaminase, soluble	0.482968	0.014529
Gsta4	Glutathione S-transferase, alpha 4	0.541488	0.025158
Gstm2	Glutathione S-transferase, mu 2	0.71847	0.008982
Cox8b	Cytochrome c oxidase, subunit VIIIb	0.32511	0.003044
Cox6c	Cytochrome c oxidase subunit Vic	2.011121	0.022585
**IMMUNE RESPONSE TO EXTRACELLULAR STIMULUS**			
C4b	Complement component 4B	0.6	0.041924
B2m	Beta-2 microglobulin	0.717475	0.0322
Tap2	Transporter 2, ATP-binding cassette, sub-family B	0.634635	0.043325
Igtp	Interferon gamma induced GTPase	0.533293	0.031471
Ifit3	Interferon-induced protein with tetratricopeptide repeats 3	0.198333	0.031219
Cd99l2	CD99 antigen-like 2	0.647073	0.043325
**RESPONSE TO HORMONE STIMULUS**			
Serpina3c	Serine peptidase inhibitor, clade A, member 3C	0.712025	0.045332
Serpina3g	Serine peptidase inhibitor, clade A, member 3G	0.388773	0.001845
Serpina3h	Serine peptidase inhibitor, clade A, member 3H	0.494143	0.013389
Serpina3n	Serine peptidase inhibitor, clade A, member 3N	0.505226	0.000321
**SMALL GTPASE MEDIATED SIGNAL TRANSDUCTION**			
Rasl11a	RAS-like, family 11, member A	0.674084	0.019568
Rasl11b	RAS-like, family 11, member B	0.573156	0.001066
Rab3d	RAB3D, member RAS oncogene family	0.72951	0.034662
Rhob	Ras homolog gene family, member B	1.666706	0.040915
Rhoj	Ras homolog gene family, member J	0.691116	0.031471
**OTHER GENES OF INTEREST**			
Aatk	Apoptosis-associated tyrosine kinase	0.564091	0.022585
Renbp	Renin binding protein	0.643495	0.031471
Aqp7	Aquaporin 7	0.565265	0.003405
Bmp1	Bone morphogenetic protein 1	0.634635	0.034934
Bst2	Bone marrow stromal cell antigen 2	0.334250	0.013389
Cenpa	Centromere protein A	1.455989	0.022585
Dbp	D site of albumin promoter binding protein	0.645728	0.043573
Enpp5	Ectonucleotide pyrophosphatase/phosphodiesterase 5	0.732550	0.0322
Fhod1	Formin homology 2 domain containing 1	1.371733	0.041924
Fkbp5	FK506 binding protein 5	1.215879	0.036674
Gas6	Growth arrest-specific 6	0.468785	0.037595
Gatad1	GATA zinc finger domain containing 1	1.747145	0.003405
Gtf3c5	General transcription factor IIIC, polypeptide 5	1.264003	0.0322
Hist1h2ah	Histone cluster 1, H2ah	1.634670	0.040326
Hp	Haptoglobin	0.657015	0.030981
Hspb8	Heat shock 22kDa protein 8	0.668963	0.022585
Ifi27	Interferon, alpha-inducible protein 27	0.309497	0.03069
Ift20	Intraflagellar transport 20 homolog	1.485552	0.031471
Itih4	Inter-alpha (globulin) inhibitor H4	0.243332	0.000025
Klc1	Kinesin light chain 1	1.278985	0.035963
Matn4	Matrilin 4	0.641712	0.031471
Mettl7B	Methyltransferase like 7B	0.704660	0.048333
Mid1ip1	MID1 interacting protein 1	0.720464	0.045125
Mpp1	Membrane protein, palmitoylated 1, 55kDa	0.744322	0.041924
Nasp	Nuclear autoantigenic sperm protein (histone-binding)	1.336074	0.048719
Nup107	Nucleoporin 107kDa	0.657927	0.029465
Pde1b	Phosphodiesterase 1B, calmodulin-dependent	1.522033	0.013389
Pde4dip	Phosphodiesterase 4D interacting protein	0.654289	0.038052
Prelp	Proline/arginine-rich end leucine-rich repeat protein	0.677362	0.034934
Psmc5	Proteasome (prosome, macropain) 26S subunit, ATPase, 5	1.387030	0.029465
Pxmp2	Peroxisomal membrane protein 2, 22 kDa	0.660669	0.033282
Rdbp	RD RNA binding protein	1.263127	0.045125
Rps24	Ribosomal protein S24	1.791291	0.022585
S100A13	S100 calcium binding protein A13	1.580082	0.022585
Aimp1	AminoacyltRNAsynthetase complex-interacting multifunctional protein 1	1.354724	0.025849
Slc5a6	Solute carrier family 5 (sodium-dependent vitamin transporter), member 6	0.756283	0.048936
Thrsp	Thyroid hormone responsive	0.264621	0.001714
Tk1	Thymidine kinase 1, soluble	1.629015	0.022786
Tmed4	Transmembrane emp24 protein transport domain containing 4	0.777007	0.045125
Tmem43	Transmembrane protein 43	0.637722	0.022585
Tmem45B	Transmembrane protein 45B	0.718470	0.041924
Tpm2	Tropomyosin 2 (beta)	1.418140	0.041924
Trafd1	Traf-type zinc finger domain containing 1	0.692074	0.004184
Uba7	Ubiquitin-like modifier activating enzyme 7	0.668037	0.048719
Uck2	Uridine-cytidine kinase 2	1.286097	0.019568
Uhrf1	Ubiquitin-like with PHD and ring finger domains 1	1.420107	0.043072
Usp18	Ubiquitin specific peptidase 18	0.238489	0.029154
Yeats4	YEATS domain containing 4	0.503128	0.019128
